# TNF-α and IFN-γ Together Up-Regulates Par-4 Expression and Induce Apoptosis in Human Neuroblastomas

**DOI:** 10.3390/biomedicines6010004

**Published:** 2017-12-26

**Authors:** Ganesh V. Shelke, Jayashree C. Jagtap, Dae-Kyum Kim, Reecha D. Shah, Gowry Das, Mruthyunjaya Shivayogi, Radha Pujari, Padma Shastry

**Affiliations:** 1 National Centre for Cell Science, Savitribai Phule Pune University, Ganeshkhind, Pune 411007, India; ganesh.shelke@gu.se (G.V.S.); jcjagtap@nccs.res.in (J.C.J.); shahricha.8@gmail.com (R.D.S.); gowrydas@gmail.com (G.D.); 2 Donnelly Centre, University of Toronto, Toronto, ON M5S 3E1, Canada; daekyum.kim@utoronto.ca; 3 Lunenfeld-Tanenbaum Research Institute, Mt. Sinai Hospital, Toronto, ON M5G 1X5, Canada; 4 Modern College, Ganeshkhind, Pune 411016, India; mruthyunjay@gmail.com; 5 Rasayani Biologics Pvt Ltd, 48/7, Mhalunge—Nande Road, Mhalunge, Pune 411045, India; radhapujari@gmail.com

**Keywords:** apoptosis, Par-4, NF-κB, IFN-γ, TNF-α, neuroblastoma

## Abstract

The objective of this study was to examine the combined effect of Interferon-gamma (IFN-γ) and Tumor Necrosis factor-alpha (TNF-α) on cytotoxicity and expression of prostate apoptosis response-4 (Par-4) and Par-4 interacting proteins B-cell lymphoma (Bcl-2), nuclear factor kappa-light-chain-enhancer of activated B cells/p65 subunit (NF-κB/p65), Ak mouse strain thymoma (Akt) in human neuroblastoma (NB) cells. Materials and methods included human neuroblastoma cell lines-SK-N-MC, SK-N-SH, and SH-SY5Y, which were treated with IFN-γ and TNF-α individually, or in combination, and were assessed for viability by tetrazolium (MTT) assay. Apoptosis was monitored by hypodiploid population (by flow cytometry), DNA fragmentation, Poly (ADP-ribose) polymerase (PARP) cleavage, and caspase-8 activity. Transcript level of Par-4 was measured by RT-PCR. Protein levels of Par-4 and suppressor of cytokine signaling 3 (SOCS-3) were assessed by immunoblotting. Cellular localization of Par-4 and p65 was examined by immunofluorescence. Unbiased transcript analysis for IFN-γ, TNF-α, and Par-4 were analyzed from three independent clinical datasets from neuroblastoma patients. In terms of results, SK-N-MC cells treated with a combination of, but not individually with, IFN-γ and TNF-α induced apoptosis characterized by hypodiploidy, DNA fragmentation, PARP cleavage, and increased caspase-8 activity. Apoptosis was associated with up-regulation of Par-4 mRNA and protein expression. Immunofluorescence studies revealed that Par-4 was localized exclusively in cytoplasm in SK-N-MC cells cultured for 24 h. but showed nuclear localization at 48 h. Treatment with IFN-γ and TNF-α together enhanced the intensity of nuclear Par-4. In gene expression, data from human neuroblastoma patients, levels of IFN-γ, and TNF-α have strong synergy with Par-4 expression and provide good survival advantage. The findings also demonstrated that apoptosis was associated with reduced level of pro-survival proteins–Bcl-2 and Akt and NF-κB/p65. Furthermore, the apoptotic effect induced by IFN-γ-induced Signal Transducer and Activator of Transcription-1(STAT-1), and could be due to down-regulation of suppressor of cytokine signaling-3 (SOCS3). The study concludes that a combinatorial approach using IFN-γ and TNF-α can be explored to maximize the effect in chemotherapy in neuroblastoma, and implies a role for Par-4 in the process.

## 1. Introduction

Neuroblastoma (NB) is the most common extracranial solid tumor in children and accounts for 8 to 10% of pediatric tumors [1]. Neuroblastoma is characterized by high rate of spontaneous regression suggesting activation of an apoptotic/differentiation program; however, advanced stages are associated with poor prognosis and resistance to chemotherapy [2]. Despite advancements in conventional therapies and the development of a recent line of treatments that include blood stem cell transplantation, differentiation therapy with retinoic acid, and passive immunotherapy with anti-disialoganglioside (GD2) antibodies, there is no significant improvement in the survival rate [3].

Tumor necrosis factor-alpha (TNF-α) is a pleiotropic cytokine with multifaceted functions. In vitro and in vivo studies have demonstrated strong anti-tumor activity of TNF-α in different types of cancers [4,5,6]. TNF-α has been exploited for potentiation of cytotoxicity induced by chemotherapeutic drugs. Interferon-gamma (IFN-γ) is a pro-inflammatory cytokine belonging to the type II class of interferons. The biological activity of IFN-γ as an anticancer agent is attributed to inhibition of proliferation, induction, and modulation of gene expression mediated by activation of Janus Kinase/Signal Transducer and Activator of Transcription (JAK/STAT) signaling pathway. Several earlier studies have demonstrated the effectiveness of the combination of IFN-γ and TNF-α in inducing apoptosis and necrosis in cancer cells [7,8,9,10,11,12]. However, the mechanisms underlying the combined effect of these cytokines are not clearly understood.

Prostate apoptosis response-4 (Par-4) is a pro-apoptotic protein and is highly conserved in vertebrates. Par-4 is a major player in the survival of cancer cells and down-regulation of Par-4 is documented in many types of cancers [13,14,15]. We have previously shown that Par-4 sensitized glioma stem cells and enhanced drug-induced apoptosis human glioblastoma cells [16]. This study suggested that constitutive Par-4 level by itself in not sufficient for induction of cell death but enhanced expression in response to apoptotic stimuli results in apoptosis [17,18]. The overexpression of Par-4 sensitizes cancer cell lines and tumor cells to cytotoxicity triggered by anticancer drugs such as doxorubicin, 5-Fluorouracil (5-FU) and by stimuli including TNF-α, TNF-related apoptosis-inducing ligand (TRAIL) [19,20,21]. Par-4 localizes in the nucleus in most cancer types and nuclear entry is essential for direct apoptosis [22]. Recent findings indicate that extracellular Par-4 induces cell-specific apoptosis by interaction with the cell-surface receptor glucose-regulated proteins 78(GRP78) and P53 dependent manner [23,24], suggesting a role of Par-4 in cellular-cross talk. The pro-apoptotic function of Par-4 is affected by its binding via the leucine zipper domain to several proteins including Ak mouse strain thymoma/protein kinase C-zeta (Akt/PKC-ζ), nuclear factor kappa-light-chain-enhancer of activated B-cells/p65 subunit (NF-κB/p62), wilms tumor-1(WT1), DAPk like kinase/Zipper interacting protein kinase (DLk/ZIP), Thanatos-associated domain-containing apoptosis-associated protein 1 (THAP-1), and thus blocking pro-survival pathways [25,26,27,28].

In this study, we examined the individual and combined effect of IFN-γ and TNF-α on cell death and expression of Par-4, along with its interacting proteins in human neuroblastoma cells. We show that co-treatment, but not individual treatment, of IFN-γ and TNF-α induced cytotoxicity in neuroblastoma cell lines. The apoptosis was associated with up-regulation and nuclear localization of Par-4, decreased levels of pro-survival protein, B-cell lymphoma (Bcl-2), and activation of NF-κB and Akt. The apoptotic effect was partially mediated by IFN-γ-induced STAT-1, caused by down-regulation of Suppressor of cytokine signaling-3 (SOCS3). Finally, we identified similar synergistic patterns of IFN-γ and TNF-α expression with Par-4 transcripts in human neuroblastoma patients.

## 2. Materials and Methods

### 2.1. Reagents and Chemicals

The following primary antibodies were purchased from Santa Cruz Biotechnology, Santa Cruz, CA, USA: rabbit anti-PARP (sc-7150), rabbit Par-4 (sc-1807), goat anti-Akt 1/2 (sc-1619), rabbit p-NF-κB/p65 (sc-101749) (1). Rabbit NF-κB/p65 (#4764), mouse p-STAT-1 Tyr701 (#9171), and rabbit p-Akt, Ser 473(#9271) was from Cell Signaling Technology, Danvers, MA, USA. Mouse Bcl-2 (#551109) was from BD Bioscience San Jose, CA, USA, rabbit SOCS-3 was procured from Abcam, Cambridge, MA, USA, and mouse actin from MP-Biomedicals. All common chemicals were purchased from Sigma chemicals, St. Louis, MO, USA.

### 2.2. Cell Lines

Human Neuroblastoma cells SK-N-MC, SH-SY-5Y, and SK-N-SH were obtained from American Type Culture Collection (ATCC) and cultured in Eagle’s Minimum Essential Medium MEM (E) containing 10% Fetal Calf Serum (FCS) (Gibco BRL, Carlsbad, CA, USA) and penicillin/streptomycin (Sigma, St. Louis, MO, USA) at 37 °C and 5% CO_2_.

### 2.3. Cell Viability Assay

Cells (1 × 10^4^ cells/well) seeded in 96-well plates for 24 h were treated with serial concentrations of recombinant human IFN-γ and TNF-α (BD Pharmingen, San Jose, CA, USA) alone or in combination for 24 h and 48 h. After treatment, cells were incubated with 5 mg/mL 3-(4,5-dimethylthiazol-2-yl)-2,5-diphenyltetrazoliumbromide (MTT) for 4 h, and subsequently solubilized in Dimethyl sulfoxide (DMSO). The absorbance was measured at 570 nm using a microplate reader (Molecular Devices, SPECTRA max 250, Sunnyvale, CA, USA). Readings in the untreated control cells were considered 100%.

### 2.4. Flow Cytometry Analysis

SK-N-MC cells (0.5 × 10^6^ cells/6-well plate) were treated with IFN-γ (10 ng/mL) and TNF-α (20 ng/mL) alone or in combination for 48 h and assessed for apoptosis. Control and treated cells were harvested, washed in cold Phosphate buffered saline (PBS), and fixed in 70% ethanol and washed in PBS. The cells were centrifuged and the pellet was incubated with RNase-A (5 mg/mL) (Amersham Biosciences, Piscataway, NJ, USA) for 15 min followed by propidium iodide (50 µg/mL) for 2 h at 37 °C. DNA content was determined on the FL-2A channel with a flow cytometer (FACS Vantage, Becton Dickinson, San Jose, CA, USA) equipped with a 488 nm argon laser. Ten thousand events were scored for each sample and data was analyzed using CellQuest Pro software (BD Pharmingen, San Jose, CA, USA) for the analysis.

### 2.5. DNA Fragmentation Assay 

DNA fragmentation assays were performed to determine the apoptosis on combination treatment. SK-N-MC cells (5 × 10^6^) were treated with combination of TNF-α (20 ng/mL) and IFN-γ (10 ng/mL) for 48 h. DNA was isolated, and DNA fragmentation was assessed (Suicide-Track™ DNA Ladder Isolation Kit Cat # AM41, Calbiochem, San Diego, CA, USA) and loaded on a 1.5% agarose gels. The gels were stained with 0.5 μg/mL ethidium bromides for 15 min and visualized using a gel-documentation system.

### 2.6. Western Blot Analysis

SK-N-MC cells were harvested, washed twice with PBS, and lysed in Radio-immunoprecipitation assay (RIPA) buffer (Upstate #20-188) (0.5 M Tris-HCl, pH 7.4, 1.5 M NaCl, 2.5% deoxycholic acid, 10% NP-40, 10 mM Ethylenediaminetetraacetic acid (EDTA) and protease inhibitor cocktail (Roche Diagnostics, Meyland, France), followed by centrifugation at 12,000 rpm for 30 min at 4 °C. Cells were lysed and nuclear fraction was isolated using NE-PER extraction reagent (Pierce, Thermo Fisher Scientific, Waltham, MA, USA). Supernatants were subjected to sodium dodecyl sulfate polyacrylamide gel electrophoresis (SDS-PAGE) and proteins transferred onto Polyvinylidene fluoride (PVDF) membranes. Membranes were blocked with 5% bovine serum albumin in Tris-buffered saline (TBS) containing 0.1% Tween-20 and probed with the following primary antibodies. The probed proteins were detected by the Enhanced chemiluminescence system (Pierce, Thermo Fisher Scientific, Waltham, MA, USA) according to the manufacturer’s instructions. The expression of the proteins was normalized with respect to actin.

### 2.7. Immunofluorescence Microscopy

For immunofluorescence, control and treated cells were fixed with 3.7% paraformaldehyde at room temperature for 10 min, permeabilized for 5 min with 0.2% triton X-100, and washed and blocked for 1 h in 3% Bovine serum albumin (BSA). Incubation with primary antibodies was done for 2 h at RT. After three PBS washes, the cover slips were incubated with goat anti-rabbit Cy3 (1:250, Molecular Probes, Invitrogen, Thermo Fisher Scientific, Waltham, MA, USA) antibody at RT for 60 min. Cells were stained with DAPI (Sigma, St. Louis, MO, USA) and cover slips were mounted using anti-fade mounting reagent and observed under confocal laser scanning microscope (Carl Zeiss, Jena, Germany).

### 2.8. Semi-Quantitative Reverse Transcription Polymerase Chain Reaction (RT-PCR)

Total RNA was extracted from SK-N-MC cells using Trizol. Complementary-DNA was prepared from 3 µg of total RNA by reverse transcription with MMLVRT enzyme (Life Technologies, Thermo Fisher Scientific, Waltham, MA, USA) at 37 °C for 60 min. The Par-4 transcripts of 200 bp were amplified from the cDNA using recombinant Taq polymerase (Life Technologies, Thermo Fisher Scientific, Waltham, MA, USA). Beta-actin was used as an internal control. The following primers were used:

Par-4: forward 5′GCAGATCGAGAAGAGGAAGC3′
     reverse 5′GCAGATAGGAACTGCCTGGA3′,
β-Actin: forward 5′ GTGGGGCGCCCCAGGCACC3′
     reverse 5′CTCCTTAATGTCACGCACGATTTC3′.

### 2.9. Caspase-8 Activity Assay

Caspase-8 activity was measured using a commercially available kit (FLICE assay kit, Calbiochem, La Jolla, CA, USA). Briefly, cells treated with IFN-γ (10 ng/mL), TNF-α (20 ng/mL) individually or in combination for 48 h, were washed with cold PBS, and lysed on ice in 50 µL of cold lysis buffer. Cell lysates were centrifuged at 10,000× *g* for 10 min and supernatants were collected, and assay for caspase-8 activity was performed in duplicates on a 96-well plate as per manufacturer’s protocol.

### 2.10. Gene-Expression Omnibus (GEO) Data Acquisition and Analysis

The Gene-Expression Omnibus (GEO) have functional transcriptomics data repository from microarray and next-generation sequence-based data. We analyzed microarray from three independent neuroblastoma datasets obtained from GEO (http://www.ncbi.nlm.nih.gov/geo/). The primary neuroblastoma datasets GSE45480, GSE49710, and GSE19274 with 881, 498, and 138 patients were chosen, respectively.

Expression of gene was quantile normalized and processed from the raw datasets by MatLab (version 2010a). The normalized dataset and their fold change (Patient/Average) were represented as log2 expression level. Furthermore, the gene with fold change over 0.58 (50% upregulated) or under 0.58 was defined as differentially expressed genes. For one-to-one gene correlation, MatLab (2010a) software was used and Pearson correlation analysis was performed. Fisher’s exact test was used to determine the synergy between the expressions of IFN-γ, and TNF-α with Par-4. Further, normalized expression levels IFN-γ, TNF-α, and PAR-4 in neuroblastoma patients between survival and non-survival group were measured. For this analysis, we performed Dunn’s multiple comparison test between the groups.

### 2.11. Statistical Analysis

Data are represented as mean ± standard deviation and analyzed with Sigma Stat software (Jandel Scientific, San Rafael, CA, USA). Treated cells and the corresponding controls were compared using one-way analysis of variance (ANOVA), followed by Student-Newman-Kuels test. *p* < 0.05 were considered significant.

## 3. Results

### 3.1. Neuroblastoma (NB) Cell Lines Sensitive to Combination of IFN-γ and TNF-α Induced Cell Death

**Figure 1:**
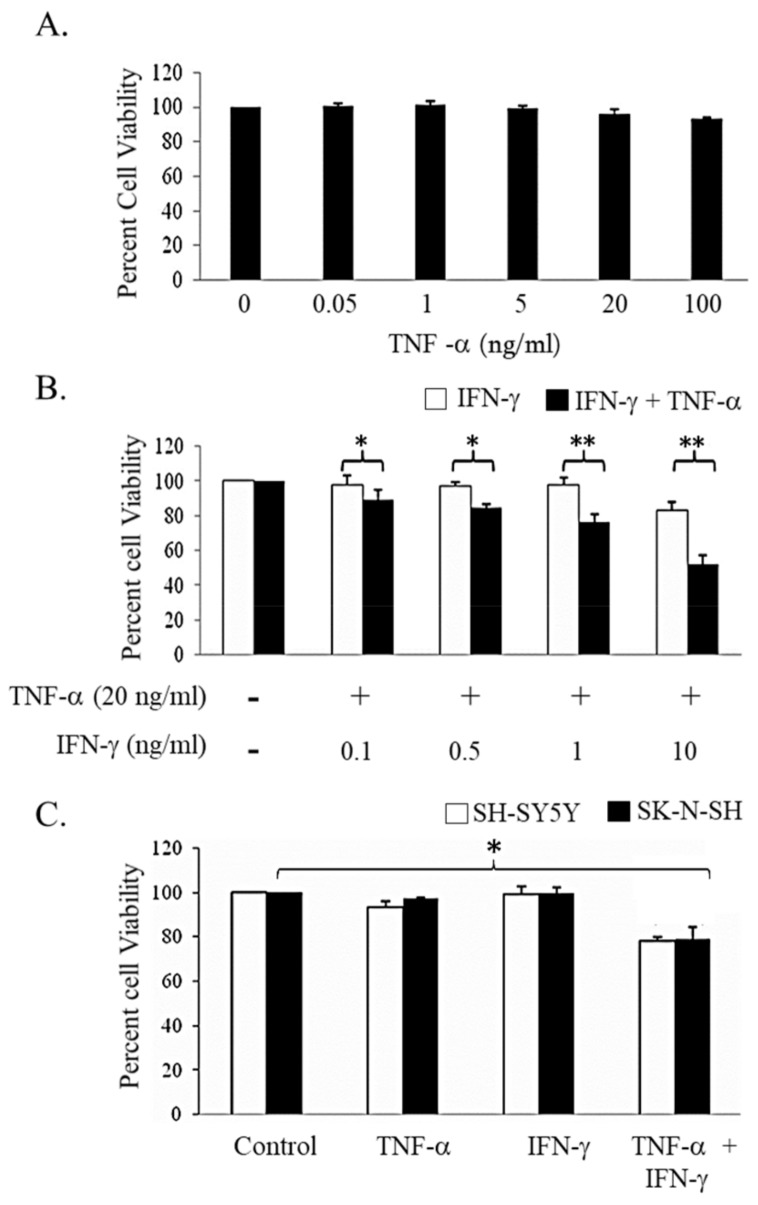
Dose-dependent effect of Interferon-gamma (IFN-γ) and Tumor necrosis factor-alpha (TNF-α) alone or in combination treatment on neuroblastoma cell lines. SK-N-MC cells were treated for 48 h with increasing concentrations of (**A**) TNF-α; and (B) IFN-γ alone and in combination and the viability was quantified by 3-(4,5-dimethylthiazol-2-yl)-2,5-diphenyltetrazolium bromide (MTT) assay. The effect of combination treatment was tested on other Neuroblastoma cell lines; SH-SY-5Y and SK-N-SH (C). Cells were treated with TNF-α (20 ng/mL) and IFN-γ (10 ng/mL) alone and in combination for 48 h and the viable cells were quantified by MTT assay. The data represented is the mean ± standard error of mean (*n* = 5). * *p* < 0.05; ** *p* < 0.005. −: untreated; +: treated.

The effect of IFN-γ and TNF-α alone and in combination on cell viability in human NB cell line SK-N-MC was evaluated by MTT assay. As depicted in Figure 1A, the cells were resistant to TNF-α. Treatment with IFN-γ (10 ng/mL) decreased the cell viability by 17.3% and this was further decreased by 37.8% on treatment with TNF-α (20 ng/mL) for 48 h (Figure 1B). We also determined the effect of these cytokines in other NB cell lines. Neither, TNF-α (20 ng/mL) nor IFN-γ (10 ng/mL) individually had any effect on viability in SK-N-SH and SH-SY-5Y cell lines, but co-treatment with these cytokines for 48 h resulted in significant decrease in viability (Figure 1C). Longer exposure (72 h) of these cytokine did not reduce the viability further of SH-SY-5Y (Figure S1). These preliminary results suggested the possibility that NB cells can be sensitized by combination treatment of IFN-γ and TNF-α towards cell death. Further studies to understand the mechanism of action were performed in SK-N-MC cells.

### 3.2. Combination Treatment of IFN-γ (Interferon Gamma) and TNF-α Induced Apoptosis in SK-N-MC Cells

**Figure 2:**
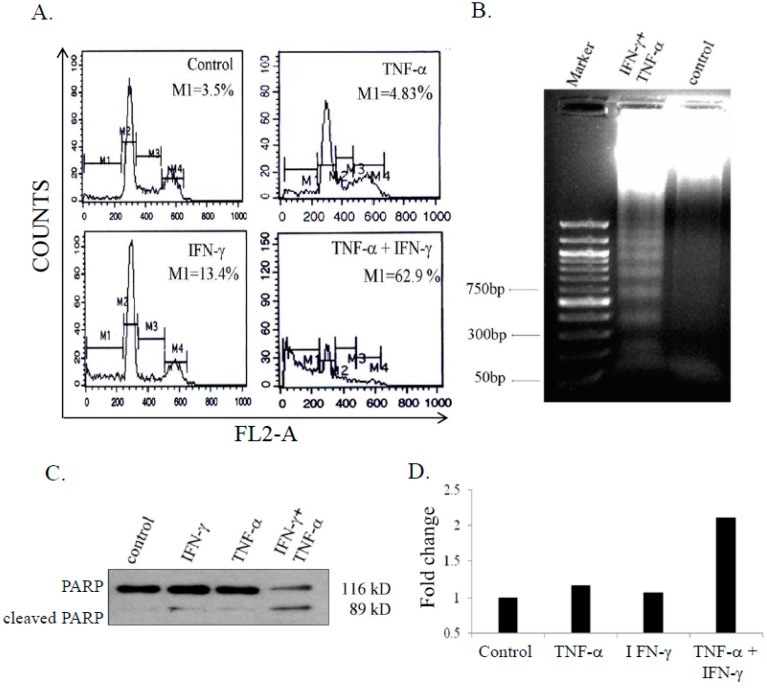
Combination of IFN-γ (interferon gamma) and TNF-α (tumor necrosis factor-alpha) enhances apoptosis in SK-N-MC cells. Cells were treated with IFN-γ (10 ng/mL) and TNF-α 20 ng/mL) alone and in combination for 48 h, and analyzed for apoptosis: (**A**) hypodiploid population by flow-cytometry; (**B**) DNA fragmentation; (**C**) Poly (ADP-ribose)polymerase (PARP) cleavage by immunoblotting; and (**D**) Casapase-8 activity assay. The figures are representative of 2 similar experiments.

To assess whether cell death induced by combination of IFN-γ and TNF-α was by apoptosis, the population of cells in sub-G1 phase were measured by flow cytometric analysis. Co-treatment with IFN-γ (10 ng/mL) and TNF-α (20 ng/mL) resulted in significant number of cells in the sub-G1 phase (62.9%) and decreased populations in G1, S, and G2/M phases. IFN-γ and TNF-α treated cells showed 13.4% and 4.83% hypodiploid cells respectively, with no significant difference in cell cycle profiles (Figure 2A). Apoptosis was also confirmed by cleavage of PARP (Figure 2B) and DNA fragmentation (Figure 2C). Activity of caspase-8 is essential for cellular apoptosis [29]. To assess caspase-8 activity, SK-N-MC cells were treated with either IFN-γ (10 ng/mL) or TNF-α (20 ng/mL) alone and in combination for 48 h. IFN-γ and TNF-α alone had no effect on caspase-8 activity, however, in combination the cytokines induced caspase-8 activity that was 2.1 fold higher compared to untreated control cells (Figure 2D).

### 3.3. Combined Treatment of IFN-γ and TNF-α Induced Expression of Pro-Apoptotic Protein-4

**Figure 3:**
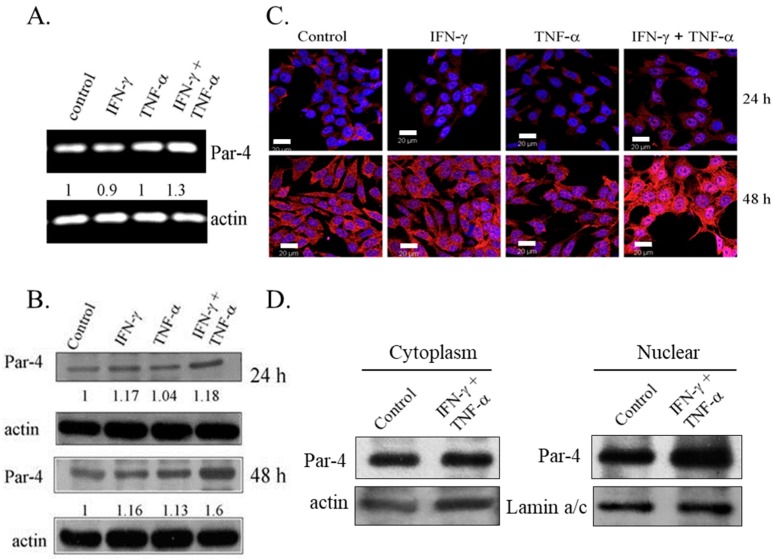
Combination of IFN-γ and TNF-α up-regulates the expression of Prostate apoptosis response-4 (Par-4). SK-N-MC cells were treated with IFN-γ (10ng/mL) and TNF-α (20 ng/mL) alone and in combination and analyzed for the expression of Par-4 by: (**A**) RT-PCR; (**B**) immunoblotting. Actin was used as loading control in both; (**C**) Immunofluorescence shows merged images with Par-4 (red) and nucleus (blue); Bar = 20 μm and (**D**) Immunoblotting represents level of Par-4 in cytoplasmic, and nuclear fraction of SK-N-MC cells at 48 h with actin and lamin-a/c as loading control, respectively.

Expression of Par-4, a pro-apoptotic protein, is absent or low in NB cell lines [30]. To examine the role of Par-4 in induction of apoptosis, the expression of Par-4 was determined in SK-N-MC cells treated with IFN-γ and TNF-α, alone and in combination. An increase in the transcripts levels of Par-4 was observed with combination treatment for 48 h (Figure 3A). Western blot analysis revealed that Par-4 was constitutively expressed in SK-N-MC cells and the expression was increased in cells cultured for 48 h compared to 24 h. Immunoblotting analysis showed that the combined treatment of IFN-γ and TNF-α up-regulated the levels of Par-4 in a time dependent manner, and was significantly higher compared to control cells and cells treated with IFN-γ and TNF-α alone (Figure 3B). Immunofluorescence studies indicated that Par-4 was localized exclusively in cytoplasm in cells cultured for 24 h. It was noteworthy that control SK-N-MC cells cultured for 48 h showed nuclear expression of Par-4, and the combined treatment of IFN-γ and TNF-α enhanced the intensity of staining of Par-4 remarkably in the nucleus (Figure 3C). This was further confirmed by increased expression of Par-4 in nuclear fraction in IFN-γ and TNF-α co-treatment (Figure 3D). Such a response is an important feature during Par-4 induced apoptosis [26].

### 3.4. Co-Treatment with IFN-γ and TNF-α Resulted in Down-Regulation of Bcl-2 and Phosphorylated Akt in SK-N-MC Cells

**Figure 4:**
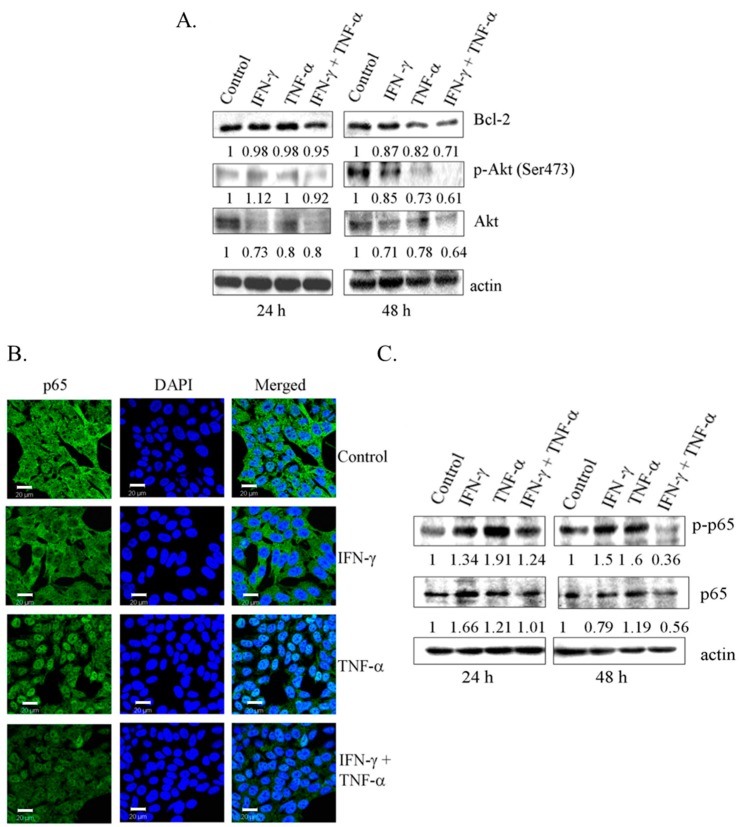
Down-regulation of anti-apoptotic proteins in SK-N-MC cells treated with the combination of IFN-γ and TNF-α. (**A**) SK-N-MC cells were treated with IFN-γ (10 ng/mL) and TNF-α (20 ng/mL) alone and in combination for 24 h and 48 h. Lysates were subjected to SDS-PAGE, followed by western blot analysis with the antibodies indicated; (**B**) SK-N-MC cells grown on glass cover slips were exposed to IFN-γ (10 ng/mL) and TNF-α (20 ng/mL) and in combination for 48 h, and analyzed for the localization NF-κB/p65 by immunofluorescence (Bar = 20 μm); and (**C**) immunoblotting analysis. Bcl-2: B-cell lymphoma-2; Ak mouse strain thymoma: Akt.

Overexpression of Par-4 is directly associated with the down-regulation of anti-apoptotic protein, Bcl-2 at protein and mRNA level [31]. Consistent with this finding, in this study, co-treatment with IFN-γ and TNF-α (24 h and 48 h) resulted in significant down-regulation of Bcl-2 protein (Figure 4A). Also, reduction in Bcl-2 at mRNA level was observed at 48 h (Figure S2). Functional regulator of Par-4, including Akt and NF-κB/p65 in cytoplasm, can render its ability to interact with other proteins [21,25,26]. Combination treatment with IFN-γ and TNF-α decreased the levels of phosphorylated Akt (Ser-473) in SK-N-MC cells (Figure 4A). As shown in Figure 4B, TNF-α induced activation of NF-κB with nuclear translocation of p65 subunit. Interestingly, while IFN-γ alone had no effect on p65, it inhibited the expression of nuclear p65 induced by TNF-α. Western blot data using antibody to phosphorylated p65 confirmed this observation (Figure 4C). These results demonstrated that enhanced expression of Par-4 with concomitant decrease in the pro-survival proteins Bcl-2, Akt, and NF-κB/p65 might be crucial in apoptosis induced by combined treatment of IFN-γ and TNF-α in SK-N-MC cells.

### 3.5. IFN-γ Signaling Is Critical for Induction of Apoptosis on Combination Treatment

**Figure 5:**
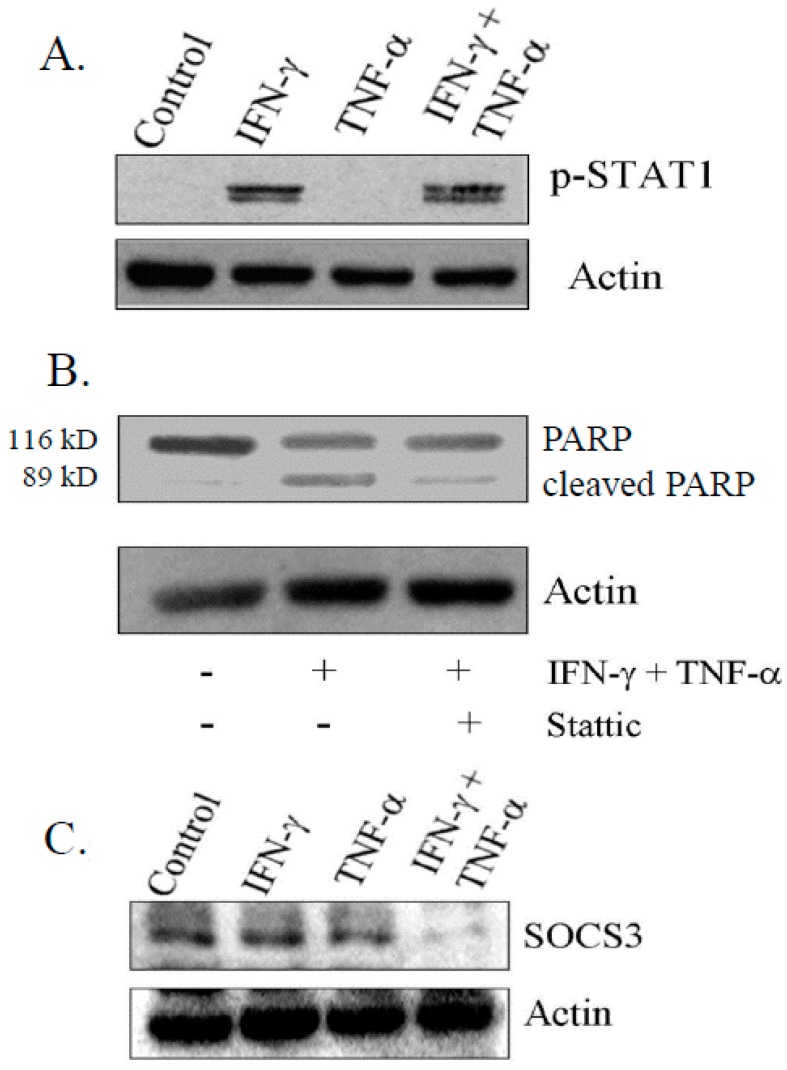
Activation of Signal Transducer and Activator of Transcription-1 (STAT1) on phosphorylation by IFN-γ alone and in combination with TNF-α. (**A**) Activation of phospho-STAT1 (Try-701) in IFN-γ and in combination with TNF-α by immunoblotting; (**B**) Decrease in total PARP in cells treated with STAT1 inhibitor (Stattic) at 2.5 µg/mL; and (**C**) Expression of suppressor of cytokine signaling-3 (SOCS3) on combination treatment for 48 h determined by immunoblotting. −: untreated; +: treated.

IFN-γ-induced apoptosis is mediated by STAT-1 signaling in a variety of cancer cells [32,33,34]. Consistent with earlier reports, we observed that the activation of STAT-1 (Tyr 701) was induced by IFN-γ, but not with TNF-α (Figure 5A). Furthermore, treatment of cells with STAT inhibitor (Stattic) (2.5 µg/mL) prior to combination treatment resulted in significant reduction of levels of 89 kDa cleaved fragment of PARP, suggesting that activation of STAT-1 was important for the induction of apoptosis (Figure 5B). A negative feedback loop regulates the cytokine signaling induced by expression of SOCS family [35]. In this context, we found a drastic reduction in SOCS-3 level in cells treated with combination, compared to control and cells exposed to IFN-γ and TNF-α alone as determined by immuno-blotting (Figure 5C).

### 3.6. TNF-α and IFN-γ Co-Expression Synergize with Par-4 (Prostate Apoptosis Response-4) Expression in Neuroblastoma Patients

**Figure 6:**
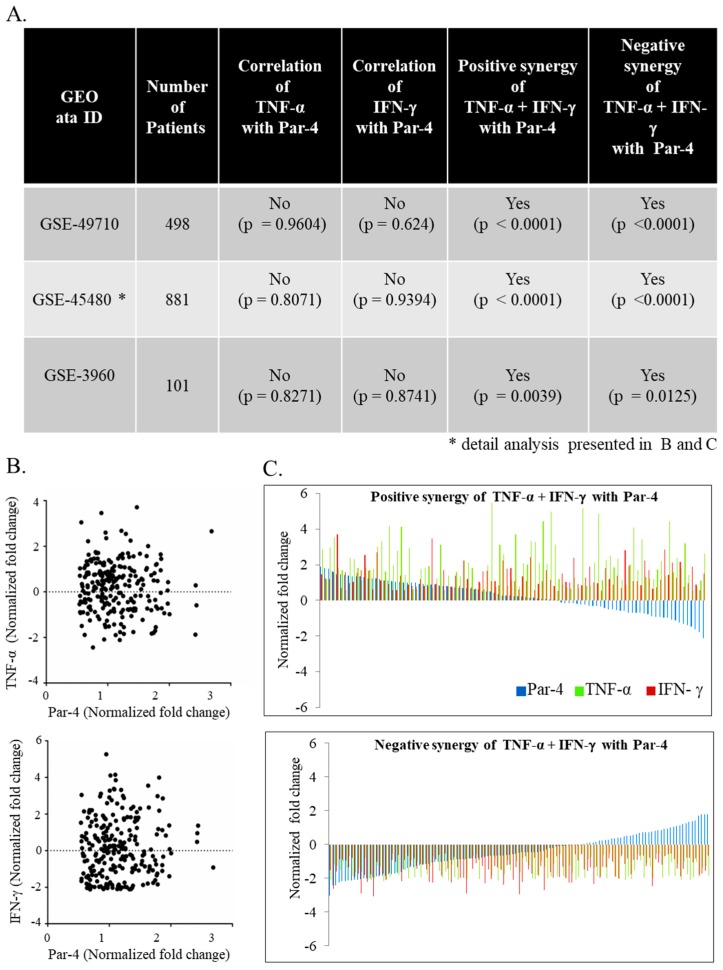
Correlative gene-expression profile of IFN-γ and TNF-α in relation with Par-4 of human neuroblastoma datasets. Three datasets from neuroblastoma patients were obtained from gene expression omnibus (GEO) database (**A**) We evaluated the individual correlation between Par-4 with IFN-γ or TNF-α and significance value described for one dataset; (**B**) positive (upper panel; also upper panel in (**C**)); and negative (lower panel; also lower panel in (**C**)) synergies were determined in the datasets by determining the correlation between differentially regulated Par-4 with IFN-γ and TNF-α together. GSE: GEO accession number; *: detail analysis presented in (**B**,**C**).

**Figure 7:**
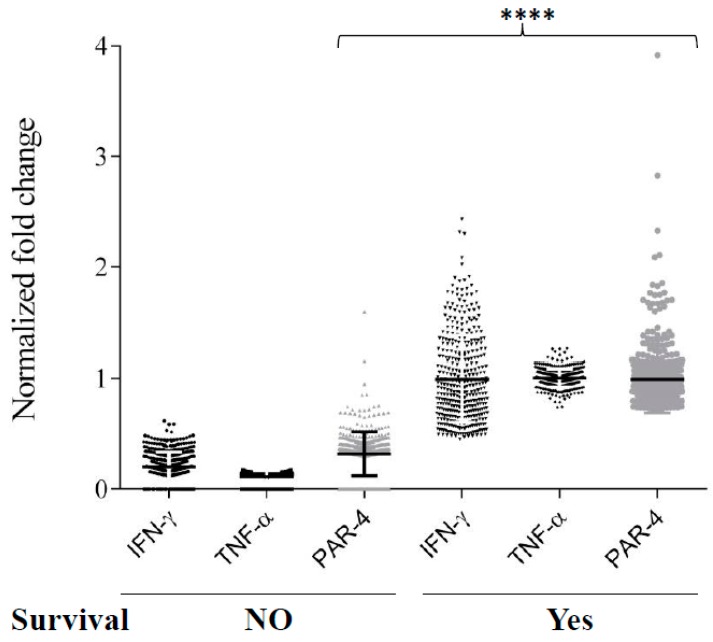
Expression levels of IFN-γ, TNF-α, and PAR-4 in neuroblastoma patients and their survival. Normalized gene expression of IFN-γ, TNF-α, and PAR-4 were analyzed in patients with their survival outcome. Dunn’s Multiples comparison was used to determine significant differences between groups (**** *p* < 0.0001).

Par-4 expression is described in neuroblastoma, but its context-dependent function makes it a poor predictor of biomarker [30]. Therefore, we sought to determine if clinical evaluation of neuroblastoma transcript could help predict the outcome of diseases identified in our invitro findings. Differential expression of Par-4 was not correlated with TNF-α or IFN-γ alone by Pearson correlation (Figure 6A)*.* Interestingly the expression of Par-4 was very strongly (*** *p* < 0.0001) correlated with expression of TNF-α and IFN-γ taken together (Figure 6B) in all three independent GEO datasets (Figure 6A). In other words, in the set of patients with either TNF-α and IFN-γ upregulated or downregulated together, we found similar regulation in Par-4 transcripts i.e., upregulated or downregulated, respectively. Upon further close inspection of selected patients with upregulated Par-4 (>0.53-fold changes), TNF-α and IFN-γ levels revealed that most of them were in stage 4 neuroblastomas with amplified myelocytomatosis gene (Myc) that was in line with a previous finding [36]. Careful analysis of normalized transcripts levels (TNF-α, IFN-γ, and Par-4) from the dataset (GSE49710) from patients with survival information was performed. Survival data (Figure 7) was strongly correlated with higher levels of Par-4 that intern relates with higher TNF-α and IFN-γ levels (Figure 6C). Taken together, this study substantially validates our in vitro in vitro finding in clinical datasets, and strongly argues that expression level of TNF-α and IFN-γ together matches with Par-4 levels.

## 4. Discussion

In the current study, we show that a combination treatment of IFN-γ and TNF-α induces apoptosis in human NB cells. Our results demonstrate that IFN-γ and TNF-α up-regulate pro-apoptotic protein-Par-4 with a concomitant decrease in the expression of pro-survival protein-Bcl-2, and activation of NF-κB and Akt. We also show that IFN-γ and TNF-α together down-regulated SOCS3. Additionally, IFN-γ induced STAT-1 that was crucial for apoptosis. Finally, we also confirm that the gene expression profile of IFN-γ and TNF-α together synergistically correlate with the expression of Par-4 in human neuroblastoma patients. Clinical gene-expression data strongly argues that the level of Par-4 was higher in the patients group, with more survival then the poor survival. Taken together, this in vitro in vitro and clinical data confirms that co-expression of IFN-γ and TNF-α enhances the expression of Par-4 that gives beneficial survival rates in Neuroblastoma patient.

Caspase-8, a key mediator of apoptosis, is activated by cell death receptor signaling pathway. Caspase-8 has been reported to be deleted frequently or silenced in neuroblastoma tumors and in cell lines including NB-7, NB-8, and NB-10 [36,37,38]. The resistance of neuroblastoma cells to apoptosis induced by TNF, tumor necrosis factor-related apoptosis-inducing ligand (TRAIL), and cytotoxic agents such as doxorubicin, has been attributed to a lack or diminished expression of caspase-8 [36,39]. Recent studies show that IFN-γ increased caspase 8 mRNA and protein, critical for cytotoxic effect of stimuli-such as TRAIL, 5 FU, and 5-Aza-2(prime)-deoxycytidin [21,39,40]. Caspase-8 activity is pre-requisite for Par-4 cleavage in TNF-α induced cell death is reported in breast cancers [29]. In this study, we found that caspases-8 activity was unaltered on exposure to IFN-γ alone, but was increased when combined with TNF-α. The discrepancy between our results with IFN-γ and the reports may be due to the difference in estimating enzyme level versus activity.

The pro-apoptotic protein, Par-4 is frequently transcriptionally or post-transcriptionally down-regulated in central nervous system tumor cell lines, U87-MG, U251-MG, and human NB cell line- SH-SY5Y [30]. The intracellular localization of Par-4 is variable in different cell types [41]. We found that a SK-N-MC cell (24 h cultures) predominantly expressed Par-4 localized in cytoplasm, but enhanced nuclear expression was also observed in 48 h cultures. Similarly, Par-4 levels were found to be higher in nuclear fraction at a longer time point compared to cytoplasmic fractions. Considering the earlier report that HEC25-cells in G1 phase showed increased nuclear Par-4, it is possible that the nuclear Par-4 in SK-N-MC cells may be due to higher population of G1 cells in 48 h cultures [42]. Increased expression of Par-4 in renal carcinomas renders them sensitive to TNF-α mediated cytotoxicity and 5-fluorouracil-induced apoptosis in human colon cancer cell line, HT29 [43,44]. In this study, treatment with IFN-γ and TNF-α alone had minimal effect on Par-4 expression but a combined treatment of these cytokines increased expression of nuclear Par-4, suggesting that the treatment rendered the cells sensitive to apoptosis. This finding is consistent with the report that in cancer cells, resistance to apoptosis correlates with resistance of Par-4 to translocate to nucleus [17,18].

One of the mechanisms in Par-4 induced apoptosis involves the inactivation of transcriptional activity of NF-κB/p65 mediated by inhibition of atypical Protein kinase C [45]. In this context, we examined whether the up-regulation of Par-4 on treatment with combination of IFN-γ and TNF-α affected the NF-κB. TNF-α-induced apoptosis in SK-N-MC cells is shown to be mediated by a mechanism involving enhanced FasL expression through nuclear factor of activated T-cells (NFAT) activation [46]. In contrast to this report, in our experiments, TNF-α alone did not induce apoptosis though enhanced p65 activity. Our finding is consistent with the report that TNF-α alone is unable to induce cell death in SK-N-MC cells and IFN-γ sensitized the cells to TNF-α-induced apoptosis by inhibition of NF-κB activation [47]. Our finding also suggests that apoptosis induced by co-treatment of IFN-γ and TNF-α was triggered by induction of nuclear Par-4 and inactivation of pro-survival signaling of NF-κB.

Reports on the role of Bcl-2 in apoptosis in context with Par-4 are contradictory. While Par-4 mediated apoptosis has been demonstrated to be independent of the endogenous Bcl-2 levels in A-172, SH-SY5Y cell lines [30], others have suggested that down-regulation of Bcl-2 is crucial in Par-4 mediated apoptosis [31]. Par-4 has been shown to bind to Bcl-2 promoter through WT1 to transcriptionally down-regulate its expression [48]. In HL-60 and K562 leukemia cell lines, up-regulation of Par-4 on treatment with arsenic contributes to induction of apoptosis, rather than down-regulation of Bcl-2 [49]. We found that Bcl-2 was down regulated at RNA and protein levels in SK-N-MC cells treated with co-treatment of IFN-γ and TNF-α, suggesting that Bcl-2 might be important in apoptosis. At this point, it is not clear whether the reduced Bcl-2 level is directly dependent or not on Par-4 in SK-N-MC cells.

The PI3K/Akt pathway is a new predictor of poor outcome in neuroblastoma, indicating the significance of the pathway in chemotherapy [50]. Recent reports have provided evidence that Par-4 is negatively regulated by Akt signaling. SK-N-MC cells treated individually with IFN-γ and TNF-α led to reduced levels of both phosphorylated (ser-473) and total Akt, while a combination of these cytokines further decreased the levels, probably reflecting the additive effect. While these findings clearly show an inverse correlation of Par-4 and Akt, it also raises the possibility that Par-4 is upregulated because of down-regulation of Akt during apoptosis induced by combination of IFN-γ and TNF-α.

STAT1 is a promoter of apoptosis and sensitizes cells to chemotherapy [51]. Consistent with earlier reports, IFN-γ alone induced phosphorylation of STAT1 (Tyr 701). Also, combination treatment did not alter the level, suggesting that TNF-α is not important in STAT1 phosphorylation. SOCS3, a negative regulator of IFN-γ signaling was down regulated with combined treatment but not individual treatment of IFN-γ and TNF-α. Levels of transcripts of SOCS3 was found to be increased with increased survival of neuroblastoma with leptin suggesting its role in neuroblastoma [52]. Additionally, we observed also observed down regulation SOCS3 with reduced transcripts of Bcl-2 as reported earlier in prostate cancers cells with regards to apoptosis [35]. Further, blocking STAT1 signaling with “stattic” blocked the in apoptosis in SK-N-MC cells. These results implicate the participation of STAT1 signaling in the apoptotic process.

The effect of TNF-α and IFN-γ in regulating Par-4 function led us to investigate if they can be predictors of neuroblastoma in clinical datasets. mRNA transcript levels of TNF-α, IFN-γ, and Par-4 show high degree of synergistic behaviors across all datasets that were tested (Figure 6A). Strong enrichment (*p* < 0.0001) in Par-4 expression was observed when TNF-α and IFN-γ were considered together (Figure 6C). Interestingly, one-to-one correlation analyses fail to identify any correlation across all datasets. This data strongly argues that in neuroblastoma, expression of either one of cytokine tested (TNF-α, or IFN-γ) is not sufficient to predict the level of pro-apoptotic protein, Par-4. Our in vitro in vitro data matched with the clinical gene expression where survival outcome is strongly argues/matches with the positive synergy of Par-4 with TNF-α and IFN-γ expression (Figure 7). Moreover, we also identified that the majority of patients with positive synergy between TNF-α, IFN-γ, and Par-4 were in stage 4 neuroblastoma with amplified Myc gene expression. Such changes indicate that these patients could be undergoing chemo-/radio-therapy for stage-4 treatments.

In conclusion, the present data suggests that though human neuroblastoma cells are resistant to IFN-γ and TNF-α, a combination of these cytokines strongly exert cytotoxic effects in neuroblastoma cells. The sensitization was associated with induction of pro-apoptotic protein Par-4 and inhibition of NF-κB, Bcl-2, and Akt. The study also underscores the significance of STAT1 activation by IFN-γ in induction of apoptosis by the cytokine combination. Finally, this study also confirms the invitro finding in clinical datasets, and provides evidence that positive synergy of TNF-α and IFN-γ together with Par-4 is a strong predictor of advance stage neuroblastoma. However, further in-depth mechanistic studies are warranted to define the precise role of IFN-γ and TNF-α signaling for eliciting apoptotic signals.

## Supplementary Materials

The following are available online at www.mdpi.com/2227-9059/6/1/4/s1, Figure S1: Viability of SH-SY-5Y cells after 72 h exposure of IFN-γ and TNF-α alone and in combination treatment, Figure S2: Analysis of Bcl-2 transcript using RT-PCR at 24 and 48 h time on exposure of IFN-γ and TNF-α alone and in combination.
