# Supplementary material for: TNF-α and IFN-γ Together Up-Regulates Par-4 Expression and Induce Apoptosis in Human Neuroblastomas
Source: Biomedicines. 2017 Dec 26;6(1):4. doi: 10.3390/biomedicines6010004 (PMC5874661; doi:10.3390/biomedicines6010004)
Supplement: Supplementary file 1 [file biomedicines-06-00004-s001.pdf]

Supplementary Materials: TNF-α and IFN-γ together up-regulates Par-4 expression and induce apoptosis in human neuroblastomas

Ganesh V. Shelke, Jayashree C. Jagtap, Dae-Kyum Kim, Reecha D. Shah, Gowry Das, Mruthyunjaya Shivayogi, Radha Pujari and Padma Shastry

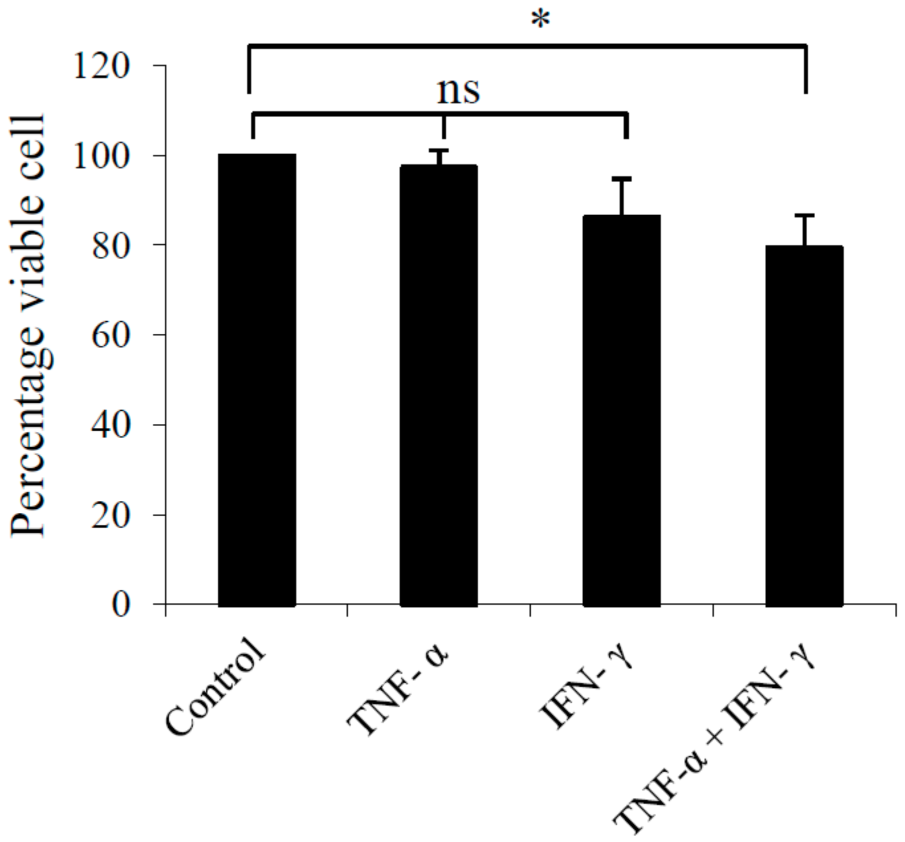

Figure S1. Viability of SH-SY-5Y cells after long exposure of Interferon-gamma (IFN-γ) and Tumor necrosis factor-alpha (TNF-α) alone and in combination treatment. Cells were treated with TNF-α (20 ng/ml) and IFN-γ (10 ng/ml) alone and in combination for 96 h and the viable cells were quantified by 3-(4,5-dimethylthiazol-2-yl)-2,5-diphenyltetrazolium bromide (MTT) assay. Data represent mean ± SD ($n = 3$). * $p < 0.05$; ns: no significant difference .

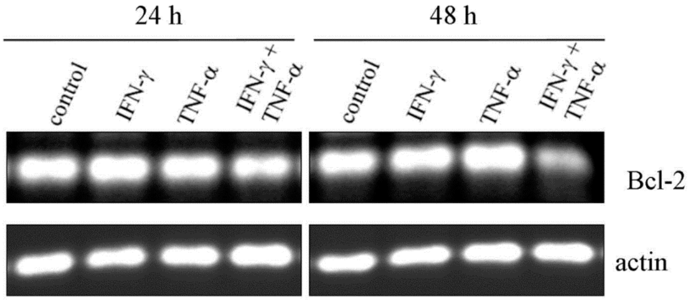

Figure S2. Analysis of B-cell lymphoma-2 (Bcl-2) transcript using RT-PCR at 24 and 48 h time on exposure of IFN-γ and TNF-α alone and in combination.
